# Brain Cells in the Avian ‘Prefrontal Cortex’ Code for Features of Slot-Machine-Like Gambling

**DOI:** 10.1371/journal.pone.0014589

**Published:** 2011-01-25

**Authors:** Damian Scarf, Kirby Miles, Amanda Sloan, Natalie Goulter, Matt Hegan, Azade Seid-Fatemi, David Harper, Michael Colombo

**Affiliations:** 1 Department of Psychology, University of Otago, Dunedin, New Zealand; 2 AE Biopsychologie, Fakultat fur Psychology, Ruhr-Universitat Bochum, Bochum, Germany; 3 Department of Psychology, Victoria University Wellington, Wellington, New Zealand; University of Lethbridge, Canada

## Abstract

Slot machines are the most common and addictive form of gambling. In the current study, we recorded from single neurons in the ‘prefrontal cortex’ of pigeons while they played a slot-machine-like task. We identified four categories of neurons that coded for different aspects of our slot-machine-like task. Reward-Proximity neurons showed a linear increase in activity as the opportunity for a reward drew near. I-Won neurons fired only when the fourth stimulus of a winning (four-of-a-kind) combination was displayed. I-Lost neurons changed their firing rate at the presentation of the first nonidentical stimulus, that is, when it was apparent that no reward was forthcoming. Finally, Near-Miss neurons also changed their activity the moment it was recognized that a reward was no longer available, but more importantly, the activity level was related to whether the trial contained one, two, or three identical stimuli prior to the display of the nonidentical stimulus. These findings not only add to recent neurophysiological research employing simulated gambling paradigms, but also add to research addressing the functional correspondence between the avian NCL and primate PFC.

## Introduction

In 1783, George Washington [1] depicted gambling as “the child of avarice, the brother of iniquity, and the father of mischief” and that it was a “vice…productive of every possible evil; equally injurious to the morals and health of its votaries.” Of all the forms of gambling, the one most associated with negative outcomes is the slot machine [2], [3]. In fact, slot machines are commonly referred to in both empirical studies and the media as the ‘crack cocaine’ of gambling [4]–[7]. There are several reasons for this label: when compared to other forms of gambling, slot machine gamblers are among the most frequent seekers of clinical treatment [8], [9], the latency from regular involvement in gambling to pathological gambling is shorter for those who gamble on slot machines [4], slot machine gamblers have higher rates of bankruptcy [10], and slot machine gamblers are more likely to develop psychiatric difficulties [10].

The Diagnostic and Statistical Manual of Mental Disorders, Fourth Edition, Revised (DSM-IV-R) defines pathological gamblers as those who have “persistent and recurrent maladaptive gambling behavior” [11]. Functional magnetic resonance imaging (fMRI) studies have demonstrated that pathological gamblers can be distinguished from healthy controls based on activity in the prefrontal cortex (PFC) [12]–[15]. Specifically, pathological gamblers display significantly different frontal activation when viewing gambling related videos [12], in response to received rewards [13], [14], and during decision-making processes [15]. Lesion studies also support the role of the PFC in gambling. The Iowa Gambling Task (IGT), which tests decision-making under ambiguous risky conditions, has been used extensively to study cognitive impairment in prefrontal patients. A consistent finding is that prefrontal patients make maladaptive choices and appear oblivious to the future consequences of those choices [16]–[18]. Interestingly, the performance of pathological gamblers on the IGT parallels that of frontal patients [19]–[24].

In the present study we recorded the responses of single neurons in the nidopallium caudolaterale (NCL) of homing pigeons while they played a slot-machine-like task. On the basis of behavioral, lesion, neurochemical, and anatomical research, the NCL is considered to be analogous to the PFC [25]–[31]. For example, similar to the PFC, the NCL is reciprocally connected with the visual, somatosensory, and auditory areas [26] and receives strong dopamine innervation [31]. Also, single neurons in the NCL participate in executive control, a critical component of PFC function [29]. The slot-machine-like task we used had an upwards pointing ‘arm’ that when pecked assumed a downwards position and activated four tumblers with associated rolling sounds and visual displays typically seen on any casino slot machine. The pigeons pecked at each tumbler to stop its motion. Reinforcement was delivered only when all four tumblers displayed identical stimuli. The primary aim of the present study was to identify neural correlates of slot-machine-like gambling in a nonhuman species.

## Results

### Histology

**Figure 1:**
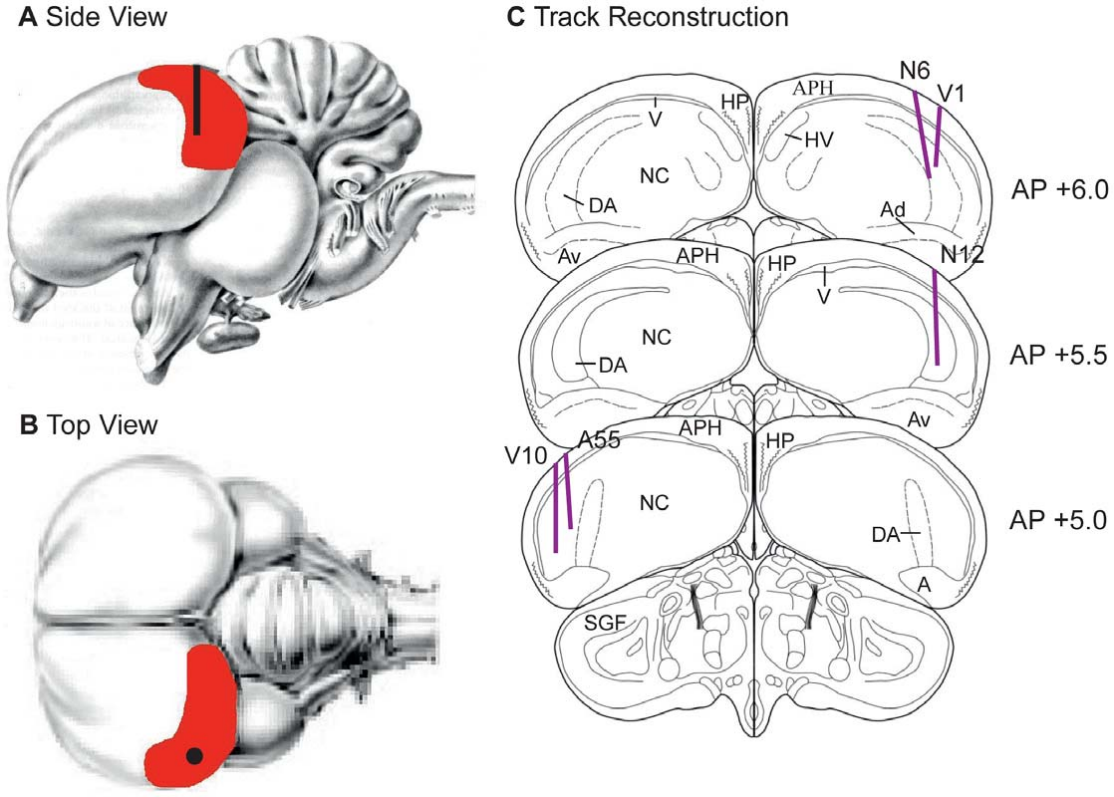
Histology. A) Lateral view of the pigeon brain. The nidopallium caudolateral (NCL) is shaded in red. The black line represents the intended electrode trajectory. B) Top view of the pigeon brain. The dot represents the intended position of the electrode entry point in NCL. C) Histological reconstruction of the electrode tracks for the five pigeons. The black lines represent the electrode track. All tracks were within the boundaries of the NCL. A: arcopallium; Ad: arcopallium dorsale; Av: arcopallium mediale; APH: area parahippocampalis; DA: tractus dorso-arcopallialis; Hp: Hippocampus; HV: mesopallium; NC: nidopallium caudale; SGF: stratum griseum et fibrosum superficiale; V: ventricle.

We recorded from a total 163 neurons across five birds. With respect to the placements of the electrodes, all tracks were within the boundaries of NCL as defined by Kröner and Güntürkün [26]. All tracks were within .5 mm of the targeted AP +5.5 placement (range AP +5.0 to AP +6.0), and all tracks were within .5 mm of the targeted ML±7.5 placements (range AP +7.0 to AP +8.0). Figure 1 shows the electrode track reconstructions for the five birds. There was no evidence of any difference in the recordings from the right and left hemispheres, the dorsal/ventral position of the electrode, or from subject to subject, and so therefore we have collapsed across these variables. In addition, we did not notice any specific anatomical localisation or clustering of our task-related neurons, nor were there any differences in the firing characteristics of these neurons. Of the 163 neurons, 55 (33.7%) displayed task related activity. The activity of the remaining neurons did not fire significantly to any task-related parameters. Across the 55 neurons we identified four categories of neurons that responded to different aspects of our slot-machine-like task: Reward-Proximity neurons, I-Won neurons, I-Lost neurons, and Near-Miss neurons. Of the 55 neurons only four responded to more than one aspect of the slot-machine-like task.

### Neural Correlates of Gambling

**Figure 2:**
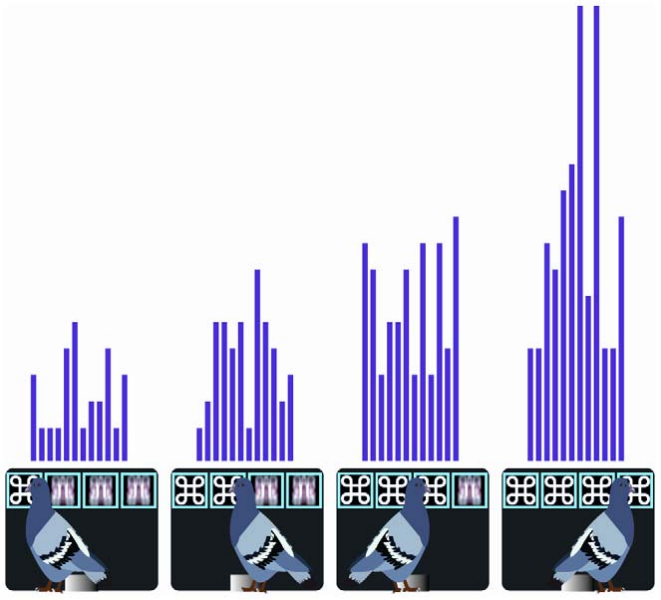
Reward-Proximity Neuron. Response profile of a Reward-Proximity neuron to each of the four tumblers on rewarded trials. The neuron shows a steady increase in firing on rewarded trials as the opportunity of a reward draws near. The period during which neural responses were measured was 300 msec, from 100–400 msec after a peck to the stimulus displayed on each tumbler. The binwidth is 25 msec. Displayed is just one of the four possible winning combinations.

A neuron was defined as a Reward-Proximity neuron if it displayed a significant linear trend in firing as the number of identical stimuli increased. Specifically, we looked to see whether a neuron increased or decreased its activity across the first two tumblers on two-of-a-kind trials, across the first three tumblers on three-of-a-kind trials, and across all four tumblers on four-of-a-kind (winning) trials. Twenty-one (12.9%) neurons displayed Reward-Proximity characteristics with 10 displaying a linear increase in activity and 11 displaying a linear decrease in activity. In all 21 Reward-Proximity neurons the linear increase or decrease was present irrespective of the particular stimuli that delivered the winning combination. An example of a Reward-Proximity neuron is shown in Figure 2. The neuron steadily increases its firing rate as the number of tumblers displaying identical stimuli increases.

**Figure 3:**
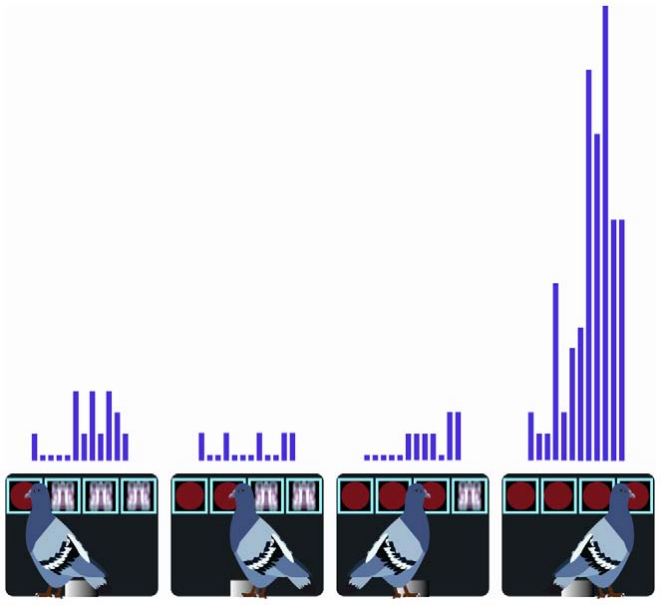
I-Won Neuron. Response profile of an I-Won neuron to each of the four tumblers on rewarded trials. The activity on the first three tumblers is no different to baseline activity, but activity to the fourth identical stimulus results in an increase in activity. The period during which neural responses were measured was 300 msec, from 100–400 msec after a peck to the stimulus displayed on each tumbler. The binwidth is 25 msec. Displayed is just one of the four possible winning combinations.

A neuron was defined as an I-Won neuron if, on winning combinations, it showed no change in activity across the first three tumblers but a significant change in activity on the final (fourth) tumbler. Seventeen (10.4%) neurons displayed I-Won characteristics. In contrast to Reward-Proximity neurons, the activity of I-Won neurons on first, second, and third tumbler was no different. Of course, the possibility exists that these neurons simply fire to the fourth tumbler irrespective of whether the tumblers display a winning or losing combination. We examined this issue by examining the activity on nonrewarded trials. For all 17 I-Won neurons, on nonrewarded trials the neural activity to the stimulus displayed on the fourth tumbler was no different than that to the stimulus on the first, second, or third tumbler. An example of an I-Won neuron is shown in Figure 3. The neuron shows very little activity to the first three tumblers but greatly increases activity when the fourth identical stimulus is displayed.

**Figure 4:**
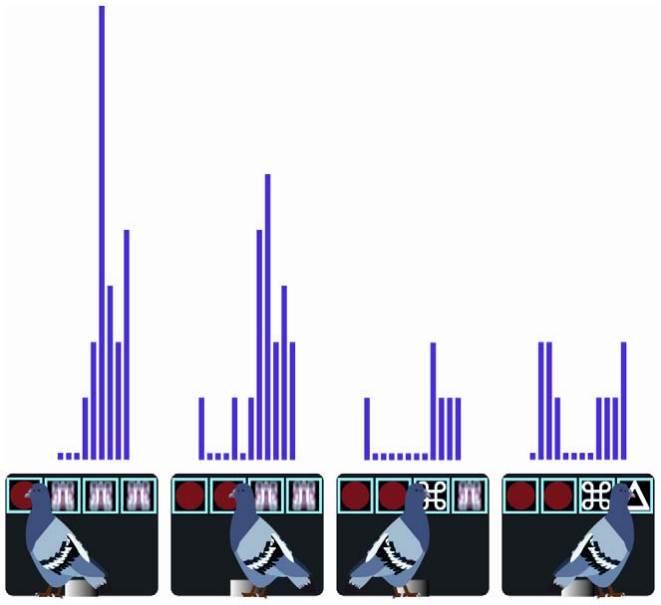
I-Lost Neuron. Response profile of an I-Lost neuron to each of the four tumblers on nonrewarded trials. The activity drops the moment it becomes apparent that no reward will be delivered. The period during which neural responses were measured was 300 msec, from 100–400 msec after a peck to the stimulus displayed on each tumbler. The binwidth is 25 msec.

A neuron was defined as an I-Lost neuron if the firing rate to the first nonidentical stimulus was significantly different to that of the preceding identical stimulus. I-Lost neurons accounted for 16 (9.8%) of the sampled neurons. These neurons fired strongly when a rewarded outcome was still possible, but reduced their firing rate at the presentation of the first nonidentical stimulus. This effect was present irrespective of whether the failure to obtain a reward occurred on the second (one-of-a-kind trial), third (two-of-a-kind trial), or fourth (three-of-a-kind trial) tumbler. An example of an I-Lost neuron is shown in Figure 4. The example shows a two-of-a-kind trial in which the nonidentical stimulus appears on the third tumbler. As the figure shows, the firing rate to the first two tumblers that display identical stimuli is high, but it then drops with the presentation of the first nonidentical stimulus.

**Figure 5:**
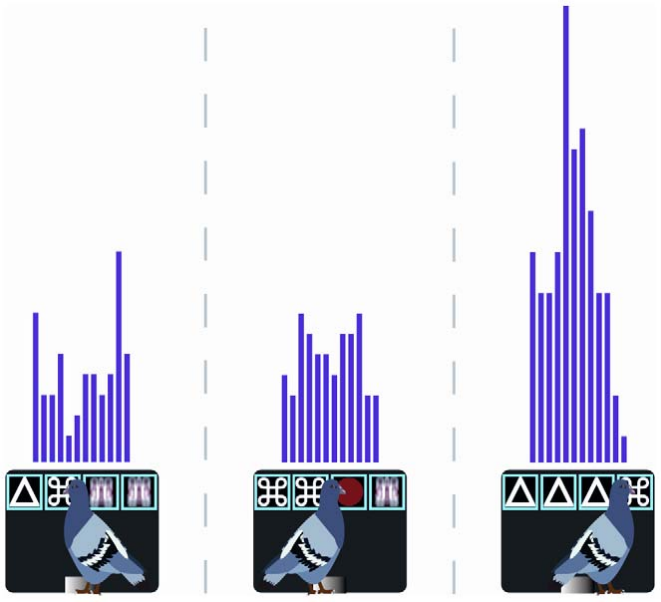
Near-Miss Neuron. Response profile of Near-Miss to the first nonidentical stimulus after one (left), two (center), or three (right) identical stimuli appearing on the tumblers. In the case of this neuron, the activity level increases as a function of the number of previous identical stimuli. The period during which neural responses were measured was 300 msec, from 100-400 msec after a peck to the stimulus displayed on each tumbler. The binwidth is 25 msec.

Finally, a neuron was identified as a Near-Miss neuron if the firing rate to the first nonidentical stimulus was significantly different to that of the preceding identical stimulus, but more importantly if the firing rate to the first nonidentical stimulus was linearly related to whether the trial contained one, two, or three identical stimuli. In other words, for a Near-Miss neuron the firing rate was related to the number of identical stimuli that appeared prior to the presentation of the first nonidentical stimulus. Near-Miss neurons accounted for 5 (3.1%) of the sampled neurons. Figure 5 shows an example of a Near-Miss neuron. In the case of this neuron, the activity to the first nonrewarded stimulus increased as a function of whether it was preceded by one (left), two (center), or three (right) identical stimuli. We believe these cells may be coding what is termed the Near-Miss effect, that is, the perception that one is closer to success the greater the number of identical stimuli that appear on a nonrewarded trial.

### Behavioral Evidence of Gambling?

A key question is whether our pigeons are performing the slot-machine-like task in a similar manner to how a person plays a casino slot machine. Unfortunately, the literature on human slot machine gambling provides very few behavioral measures of gambling which can be used for comparison. The only available data is that humans tend to display an increased latency to initiate the next trial following a rewarded trial [32]. To investigate whether our pigeons also showed a similar latency effect we compared the latencies to initiate the next trial following one-of-a-kind, two-of-a-kind, three-of-a-kind, and four-of-a-kind (winning) trials. Consistent with the human literature [32], we found that the latency following four-of-a-kind (winning) trials was longer than that following one-of-a-kind, two-of-a-kind, and three-of-a-kind trials, and that the latency following one-of-a-kind, two-of-a-kind, and three-of-a-kind trials was no different.

## Discussion

Using a slot-machine-like task we were able to identify four neural correlates of slot machine gambling in the avian NCL; Reward-Proximity neurons, I-Won neurons, I-Lost neurons, and Near-Miss neurons. Reward-Proximity neurons have been identified in the primate anterior cingulate [33], ventral striatum [34], premotor cortex [35], perirhinal cortex [36], dorsolateral PFC and orbitofrontal cortex [37], [38]. The Reward-Proximity neurons is these studies either showed differential firing across a reward schedule [33], [34], [37], [38], or significant changes between tasks that require a different number of steps to receive reward [35]. For example, Shidara and Richmond [33] had their monkeys perform a series of visual discriminations, and after a set number of discriminations the monkey was rewarded. They identified neurons in the anterior cingulate that increased or decreased their firing as the monkey drew closer to the final discrimination of a set. The Reward-Proximity neurons identified in present study differ from those identified by Shidara and Richmond [33] and the studies noted above in that they coded for the proximity to reward within a single trial rather than across several trials.

The presence of I-Won neurons indicates that the pigeons came to associate four tumblers displaying identical stimuli with a reward. Importantly, these neurons did not respond to the first, second, or third identical stimulus that preceded the presentation of the fourth identical stimulus. It was only with the appearance of the fourth identical stimulus that these neurons altered their activity levels. The fact the neurons fired after the fourth tumbler had stopped, but before the delivery of the reward, suggests they reflect the expectation that a reward would follow. Reward-expectancy neurons have been identified in the primate PFC [39]–[49]. These neurons fire during the delay following the presentation of a cue that signals reward and increase firing when a large, compared to small, reward is expected following the delay [39], [47], [48], and show differential firing patterns specific to certain types of reward (e.g. preferred vs. nonpreferred) [41], [47]. Similar anticipatory activity has been identified in the human prefrontal cortex using a range of behavioral paradigms [50]–[53]. What makes the I-Won neurons of the present study different, is that it is not a particular cue per se that resulted in the activation of these neurons, but rather the combination of cues that indicated a particular outcome, in this case a winning combination.

Because subjects did not risk anything while playing the slot-machine-like task, it is possible that they did not associate nonrewarded trials with reward omission. The fact that we were able to identify I-Lost neurons, however, supports the idea that the subjects did process nonrewarded trials as a lost opportunity for reward. These neurons fired the moment a nonidentical stimulus was presented, signalling that a reward would not be available. Previous studies have reported PFC neurons that code for the absence of a reward [43], [49], [54]. These studies, however, used specific stimuli to signal that a trial would or would not be rewarded. In the present study, the same stimuli were used on both winning and losing trials. Losing trials, therefore, could only be identified by recognising that the first nonidentical stimulus following one, two, or three identical stimuli signalled reward omission.

It has been suggested that reward-omission activity may still reflect reward processing [48], [49]. That is, although the current trial is a nonrewarded trial, the true reward may be moving to the next trial which holds the possibility of a reward. In the present study, however, although the first nonidentical stimulus signalled a nonrewarded trial, only if the nonidentical stimulus appeared on the fourth tumbler did it immediately lead to a new trial. If I-Lost neurons reflected the rewarding properties of advancing to the next trial it seems logical that the activity following the presentation of the first nonidentical stimulus on the second (one-of-a-kind trial) or third (two-of-a-kind trial) tumbler would persist for the duration of the trial. This was not the case.

Finally, although few, we did notice what we called Near-Miss neurons. A near miss is a loss that is falsely perceived as close to a win. Near-miss spins are an important property of casino slot machines. Having the correct frequency of near misses during game play has been shown to increase gamblers' persistence on a machine [55]–[58]. Recently, Clark, Lawrence, Astley-Jones, and Gray [59] completed an fMRI study that looked at the neural basis of the Near-Miss effect. They used a simulated slot machine with two tumblers. On each trial, the tumblers started rolling simultaneously and, after a variable duration, the left tumbler stopped, followed shortly after by the right tumbler. A near miss was defined as the right tumbler stopping one stop before (with the identical symbol above the pay line) or one stop after (with the identical symbol below the pay line) an identical stimulus to that displayed on the left tumbler came into the pay line. Clark et al. [59] found that compared to full misses, near misses elicited a greater BOLD signal in the ventral striatum and anterior insula, two areas that were also activated on winning spins. They concluded that the “recruitment of win-related regions during near miss outcomes underlies their ability to promote gambling behavior.”

In the present study, we defined Near-Miss neurons as those that changed activity in a linear fashion depending on whether the trial contained one, two, or three identical stimuli prior to the display of a nonidentical stimulus. We reasoned that if trials containing more identical stimuli were associated with being closer to reward, then we should see a greater change in activity upon presentation of the first nonidentical stimulus following two identical stimuli compared to one and, logically, following three identical stimuli compared to two. This is exactly what was found: the activity change of Near-Miss neurons was linearly related to whether the trial contained one, two, or three identical stimuli. Obviously, the number of Near-Miss neurons we identified (*n* = 5) was low when compared to the number of Reward-Proximity (*n* = 21), I-Won (*n* = 17), and I-Lost (*n* = 16) neurons. One potential explanation for this is the high win ratio (45.5%) used in our slot-machine-like task. It is possible that if the frequency of reward was reduced and the frequency of near misses increased (e.g. a greater number of three-of-a-kind trials) the Near-Miss neurons would be better represented. In any case, our findings both corroborate and extend those of Clark et al. [59]. The identification of Near-Miss neurons in the NCL accords well with the proposal that the effectiveness of a near miss is the result of its ability to activate areas that are also activated after a win. In addition, the fact we were able to identify neurons that convey the degree of the Near-Miss effect suggests that rather than being an all or none phenomenon [59], the Near-Miss effect is graded.

### An Analog of Human Slot Machine Gambling?

A slot machine is not a cognitively demanding task, and the ability to appreciate that four identical stimuli result in a reward, and that the first nonidentical stimulus signals a nonrewarded trial, is well within the cognitive repertoire of all vertebrates. The fact were able to find neurons in the avian NCL that coded for winning and losing components of slot machine gambling strongly suggests that our birds treated the slot-machine-like task in a similar manner to how a human plays a slot machine. Of course, there are also several aspects of the slot-machine-like task that make the analogy between the behavior of our pigeons and a person playing a slot machine difficult. Perhaps the most significant difference is that, unlike a pathological gambler, our pigeons were never behaving irrationally. Because our slot-machine-like task had a high win ratio (45.5%) and nothing was placed at risk, it was always economical for our pigeons to play. Obviously, the same is not true when a person plays a slot machine, because in the large majority of cases the person will be economically worse off after playing. As Skinner [60] noted, however, the net gain or loss is almost irrelevant when it comes to keeping a person playing a slot machine. The most important component of the slot machine, according to Skinner [60], is the schedule of reinforcement, and in this aspect our slot-machine-like task and a casino slot machine are little different [60].

### Concluding Remarks

Previous single-unit recording studies have only looked at components of gambling, such as risk [e.g. 61]. In the present study we simulated an actual gambling task and identified four neural correlates of slot machine gambling; Reward-Proximity neurons, I-Won neurons, I-Lost neurons, and Near-Miss neurons. These findings not only add to recent neurophysiological research employing simulated gambling paradigms [59], but also add to research addressing the functional correspondence between the avian NCL and primate PFC [25]–[31], [62], [63].

## Materials and Methods

### Ethics Statement

The experiment was approved by the University of Otago Animal Ethics Committee and conducted in accordance with the University of Otago's Code of Ethical Conduct for the Manipulation of Animals.

### Subjects

The subjects were five homing pigeons (*Columba livia*). The animals were housed individually in wire mesh-cages inside a colony room. The colony room was maintained on a 12:12-h light:dark cycle with the lights turned on at 7am and off at 7pm. In their home cages the pigeons were provided with water and grit ad lib. The pigeons were fed a mixture of peas, wheat, and corn in an amount adjusted to maintain them at 80-85% of their free feeding weight.

### Apparatus and Stimuli

All training and electrophysiological testing took place in a sound attenuated operant chamber. The rear and two side walls of the chamber were black. The front wall of the chamber housed an infrared touch frame that was used to register the pigeon's responses. Located behind the touch frame was a 17”computer monitor on which the stimuli were presented. The task mimicked a real slot machine with a single pay line. The stimuli were three white geometric forms (clover, triangle, and book) and a red disk. The stimuli were presented in the center of four 2.5 cm by 2.5 cm square response region arranged horizontally across the screen and all located 8 cm above the floor of the chamber. Each response region was bordered by a thin blue line representative of a tumbler. Each of the four stimuli could appear on any of the four tumblers. An arm-shaped stimulus, used to represent a slot machine arm, was also presented on the left side of the screen and was used by the pigeon to control the onset of a trial. Food reward (wheat) was delivered via an illuminated magazine centered below the four tumblers.

### Behavioral Task

All trials began with a 3-sec intertrial interval (ITI), followed by the arm-shaped stimulus assuming an upward position. A response to the arm resulted in it moving to a downward position, and thereby initiating the rolling of the four tumblers. The rolling of the tumblers was simulated, similar to a computerised slot machine, by having the four stimuli appear in a random order on each tumbler. To stop the tumblers, the birds made a single response to each of the four tumblers sequentially from left to right. That is, the birds had to peck the leftmost tumbler first, followed by second tumbler located to the right of the first, and so on until all four tumblers had been responded to. If the birds attempted to peck a tumbler without responding to the tumblers preceding it, the peck did not register and the tumbler continued to roll. When a tumbler was responded to correctly it immediately stopped rolling and displayed one of the four stimuli.

There were three losing trial types designated as one-of-a-kind, two-of-a-kind, and three-of-a-kind, and one winning trial type designated as four-of-a-kind. One-of-a-kind trials were those on which the stimulus appearing on the first tumbler was not repeated on any of the other tumblers. Two-of-a-kind and three-of-a-kind trials were those in which a stimulus was repeated across the first two and three tumblers, respectively, and then followed by different stimuli on the remaining tumblers. Finally, four-of-a-kind trials were those on which the same stimulus appeared on all four tumblers. Only four-of-a-kind trials resulted in the delivery of 3-sec access to reward, which was followed by entry into the ITI. One-of-a-kind, two-of-a-kind, and three-of-a-kind trials resulted in no reward and direct entry into the ITI. On all losing trials the bird was still required to respond to the tumblers to advance to the next trial. At the end of the ITI, the arm returned to its upward position and the pigeon was free to commence the next trial.

A session consisted of 132 trials, 24 trials of each of the three nonrewarded trial types and 60 rewarded trial types. All stimulus combinations were balanced across the four stimuli used in the experiment. Reward was delivered on an approximate Variable-Interval (VI) schedule. Casino slot machines operate on a variable ratio (VR) or random-ratio (RR) schedule. The VR and RR schedules require a large number of responses. Because of a concern for noise artefacts as a result of excessive pecking, a VI schedule was adopted. A VI schedule mimics the unpredictable nature of a VR or RR schedule without encouraging a larger number of pecks.

### Surgery

Upon completion of behavioral training, the birds were prepared for single unit recording by implanting a miniature movable microdrive [64]. Surgery was conducted under ketamine hydrochloride (100 mg/ml) and xylazine (20 mg/ml) anaesthesia. The head was immobilized using a Revzin stereotaxic adapter [65]. A topical anaesthetic (10% Xylocaine) was applied to the scalp, which was then cut and retracted to expose the skull. A small hole above the NCL was drilled through the skull at AP +5.5 and ML ±7.5 [64], and the microdrive then lowered so that the tips of the electrodes were positioned just above the NCL. Stainless steel skull screws, one serving as a ground screw, were placed into the skull, and the entire microdrive was attached to the skull using dental acrylic. The incision was then sutured, Xylocaine applied to the wound margin, and the animal allowed to recover in a heated, padded cage until alert and mobile, at which point it was returned to its home cage. All animals were allowed to recover for 7 to 14 days prior to the start of recording.

### Neuronal Recording

The microdrive housed eight 25-lm Formvar-coated nichrome wires that were used to measure the extracellular activity of single neurons. All signals were first impedance matched through a FET headstage and then amplified and filtered to remove 50 Hz noise using Grass P511K preamplifiers (Grass Instruments, Quincy, Massachusetts, United States). A separate electrode with minimal activity served as the indifferent electrode. The signals were monitored with an oscilloscope and speaker. Behavioral time-tagging of all events and analysis of the spike data was accomplished using a CED 1401 plus system (Cambridge Electronic Design Limited, Cambridge, United Kingdom) and CED Spike 2 software. The only criterion for the selection of a neuron was that it was well isolated with a signal-to-noise ratio of at least 2∶1. A typical session lasted approximately 20 to 40 min. The pigeons were tested once a day. At the end of the recording session, the electrodes were advanced at least 40 um, and the animal returned to its home cage.

### Histology and Electrode Track Reconstruction

Upon completion of the experiment, the final electrode position was marked by passing a current through each electrode, thus creating a small electrolytic lesion. The pigeons were then deeply anaesthetized with halothane and perfused through the heart with physiological saline followed by 10% formalin. The brains were blocked, removed, placed in 10% formalin for 5 d, placed in 30% sucrose and 10% formalin, and allowed to sink twice. The brains were then frozen and sectioned at 50 um, with every section mounted and stained with cresyl violet. The positions of the recorded neurons were calculated from the electrode track reconstructions, position of the electrolytic lesion, and depth records.

### Data Analysis

Pigeons reliably close their eyes just prior to and after contact with a key. Neural responses to visual stimuli were therefore analyzed for a 300 msec period from 100-400 msec after a keypeck.

Separate General Linear Model (GLM) Univariate analyses were conducted to identify the reward related (Reward-Proximity and I-Won) and loss related (I-Lost and Near-Miss) neurons. For the reward related neurons, neural responses to the first two tumblers on two-of-a-kind trials, the first three tumblers on three-of-a-kind trials, and the four tumblers on four-of-a-kind (winning) trials were analyzed together. The analysis was conducted with Trial Type (two-of-a-kind vs. three-of-a-kind vs. four-of-a-kind) and Tumbler (tumbler 1 vs. tumbler 2 vs. tumbler 3 vs. tumbler 4) as factors. Reward related neurons were identified as neurons that displayed no effect of Trial Type but a significant effect of Tumbler. The absence of an effect of Trial Type is important as it suggests that the significant effect of Tumbler was present across all three trial types. To distinguish between Reward-Proximity neurons and I-Won neurons linear trend and planed comparison analyses were conducted. A Reward-Proximity neuron was identified as a neuron that displayed a significant linear trend across tumblers. An I-Won neuron was identified as a neuron that displayed a significant change in firing rate to the fourth tumbler but for which firing to the first three tumblers was no different. The Reward-Proximity and I-Won neurons are mutually exclusive. That is, a Reward-Proximity neuron cannot be an I-Won neuron and vice versa.

For the loss related neurons, neural responses to the tumbler preceding the first nonidentical stimulus and neural responses to the tumbler displaying the first nonidentical stimulus were analyzed for one-of-a-kind trials (tumbler 1-identical and tumbler 2-nonidentical), two-of-a-king trials (tumbler 2-identical and tumbler 3-nonidentical), and three-of-a-kind trials (tumbler 3-identical and tumbler 4-nonidentical). The analysis was conducted with Trial Type (one-of-a-kind vs. two-of-a-kind vs. three-of-a-kind) and Stimulus (identical vs. nonidentical) as factors. I-Lost neurons were identified as neurons that displayed a significant effect of Stimulus but no Trial Type x Stimulus interaction. To identify Near-Miss neurons we first looked for a significant Trial Type x Stimulus interaction. After finding a significant interaction we then conducted trend analyses to determine if neural responses were linearly related to the number of identical stimuli that preceded the nonidentical stimulus. Near-Miss neurons were identified as the neurons that displayed a significant linear trend. The I-Lost and Near-Miss neurons are mutually exclusive. That is, an I-Lost neuron cannot be a Near-Miss neuron and vice versa.

To compare the response latency to start a new trial after winning (four-of-a-kind) and losing (one-of-a-kind, two-of-a-kind, and three-of-a-kind) trials, a one-way ANOVA with repeated measures across Trial Type (one-of-a-kind vs. two-of-a-kind vs. three-of-a-kind vs. four-of-a-kind) was conducted. Post-hoc Scheffe tests (evaluated at p<.05) were then used to determine if the latency following four-of-a-kind (winning) trials was longer than that following one-of-a-kind, two-of-a-kind, and three-of-a-kind trials, and that the latency following one-of-a-kind, two-of-a-kind, and three-of-a-kind trials was no different.
